# Comparative Analysis of Breast Cancer Incidence Rates between Australia and Japan: Screening Target Implications

**DOI:** 10.31557/APJCP.2020.21.7.2123

**Published:** 2020-07

**Authors:** Miwa Mia Mizukoshi, Syeda Zakia Hossain, Ann Poulos

**Affiliations:** 1 *Discipline of Behaviour and Social Sciences in Health, The University of Sydney, Australia.*; 2 *Discipline of Medical Radiation Sciences, The University of Sydney, Australia. *

**Keywords:** Breast cancer, incidence, screening, Japan, Australia

## Abstract

**Background:**

The purpose of this analysis was to compare the age-specific incidence rates (ASIRs) of breast cancer in Australia and Japan to determine the appropriateness of national screening target age groups.

**Methods:**

The paper is based on secondary sources of data. The ASIRs in 2006-2015 were collected from the Australian Institute of Health and Welfare (AIHW) and the National Cancer Center Japan. Descriptive analysis was performed for a comparison of ASIRs between Australia and Japan by age and over time. Percentage change, rolling average and risk ratio were calculated for further analysis.

**Results:**

In Australia, ASIRs rose sharply from age 40 years and peaked at 65-69 years. Japanese data demonstrated a considerable increase each year and two peaks were recorded, at ages 45-49 and 60-64. The ASIRs after age 65 decreased with age in Japan but increased with age in Australia. The ASIRs of women aged 40-49 was lowest among Australian women and the highest among Japanese women, while they had similar ASIRs in the direct comparative analysis.

**Conclusions:**

The screening age range of Australian and Japanese national breast cancer screening guidelines covers incidence peak ages in each country and therefore provides benefit for cancer screening. Our findings also indicated that further evidence is required to investigate the inclusion of Japanese migrant women in Australia aged 40-49 years into the screening target and the BCI rates of post-migrant women in Australia as different migrant groups have different ASIRs. This is to ensure that the groups of women with the highest cancer incidence are appropriately covered in screening programs.

## Introduction

Breast cancer incidence (BCI) rates indicate the number of new cases of breast cancer (Coggon et al., 2003). BCI rates vary between regions, countries and ethnicities (Seiler et al., 2017; Bray et al., 2018) and have been increasing worldwide, particularly in developing nations (Bray et al., 2018). BCI rates for a given age group and year are expressed by age-specific incidence rates (ASIR), a useful measure to determine breast cancer incidence in different age groups (Australian Institute of Health and Welfare [AIHW], 2018a). It is important for policy makers and breast cancer researchers to monitor BCI rates, especially ASIRs, as this helps to identify possible causes of increases or decreases in breast cancer incidence, influences the development of advances in treatment and, importantly, provides insight into the effectiveness of breast screening programs. 

Breast screening by mammography is currently the gold standard for the early detection of breast cancer in many countries, including Australia and Japan. Since the introduction of breast screening in Australia through the national BreastScreen Australia program in 1991, BCI rates increased from 200 to 300 per 100,000 women until 2000, but have remained steady for more than 10 years (AIHW, 2018b). In Japan, BCI rates have been increasing since 1975 and are still rising (Japanese Breast Cancer Society, 2015). The screening programs in these countries set different target age groups: in Australia, women aged 50-74 years are actively recruited while in Japan the target is women aged 40-75 (Japanese Breast Cancer Society, 2015). 

In Australia, BCI rates increased considerably from 1987-2007, mainly due to the introduction of the population-based breast cancer screening program (BreastScreen Australia) (Sitas et al., 2012) and the diagnosis of early stage cancers, including ductal carcinoma in situ (DCIS) (DeSantis et al., 2015). From 2001-2003, the decrease in the incidence of breast cancer among women aged 50 and over was seen corresponding to the decrease in the use of HRT among concession card holders, with 6.7% fewer incidence rates and 600 fewer breast cancers reported in 2003 (Canfell et al., 2008; Sitas et al., 2012). In Australia, which is a multiethnic society, BCI rates differ significantly by ethnicity (Brennan, 2017).

In Japan, the Breast cancer screening promotion project was implemented in 2009 due to an increase in BCI rates (Ministry of Health Labour and Welfare, 2015). However, the BCI rates in Japan were still only half of those in Australia between 2006-2007 (DeSantis et al., 2015). The guideline released in 2015 by the National Committee of Cancer Screening, Ministry of Health Labour and Welfare Japan indicated to recruit women aged 40 year and above (Ministry of Health Labour and Welfare, 2015) based on evidence that women in Japan have been found to develop breast cancer at a younger age compared to women in Western countries (Leong et al., 2010). Japan is an economically developed and monoethnic country in Eastern Asia that includes minority ethnic and indigenous groups, hence racial and genetic variations are considered to be minimised. 

In this study, age-specific breast cancer incidence rates (ASIRs) among Australian women and Japanese women in Japan were compared. This aims to investigate whether Australian women and Japanese women are best served by the current screening policies of Australia and Japan. 

## Materials and Methods

*Data sources*

Secondary data in the form of existing epidemiologic statistical data published by government agencies in Australia and Japan were analysed. Australian and Japanese ASIRs used in this analysis were obtained from the online sources (AIHW, 2018a; National Cancer Center Japan, 2018). The numbers of new cases per 100,000 women aged 40 and over diagnosed with breast cancer between 2006-2015 were extracted from the databases. The period of 2006-2015 was the latest decade for which data were available at the time of retrieval. The data would include both diagnostic and screening detected cases. New cases of DCIS were excluded. 

*Methods of comparative analysis*

Australian and Japanese ASIRs were plotted by 5-year age groups and by year of diagnosis for women aged over 40 years during the last 10-year period (2006-2015). Additionally, a direct comparison of incidence rates between Australia and Japan was made using national ASIRs in 2015. Screening participation and female populations in Australia and Japan were also presented by age group to explain potential contributing factors for the ASIR trends. 

Descriptive secondary data analysis was performed to provide comparative analysis of breast cancer incidence statistics in Australia and Japan. Percentage changes were calculated to observe the degree of change over time or of differences between age groups. Additionally, 5-year rolling averages were calculated to provide general trends with minimum fluctuations. The 5-year rolling average is the mean value of BCI every five years. A risk ratio (RR) was then calculated to determine the comparative risk for Japanese and Australian women to be diagnosed with breast cancer. A RR of 1 suggests no difference in incidence; > 1 suggests an increased risk; < 1 suggests a reduced risk of breast cancer.

## Results

*Age Specific Incidence Rates (ASIRs) in Australia*

**Figure 1:**
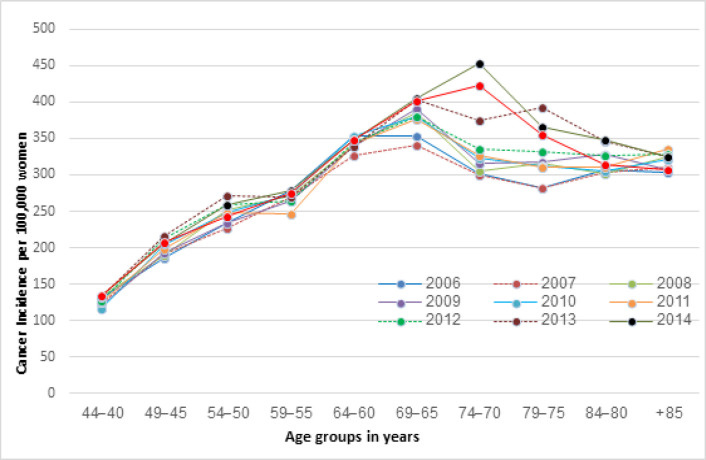
Age-Specific Incidence Rates (ASIRs) (per 100,000) among Australian Women from 2006-2015 (AIHW, 2018a)

The ASIRs for Australian women between 2006 and 2015 are shown in Figure 1. The ASIRs increased dramatically with age. There was a substantial increase in BCI among women aged from 40-44 to 45-49, and from 55-59 to 60-64 overall. The ASIR was consistently highest among women 65-69 after adjusting fluctuations by 5 year-rolling averages. Among women aged 70 years old and over, the BCI showed a slight decrease but remained high when compared to younger women, with the mean ASIRs for women aged 40-44 between 2006-2015 and women aged 80+ were 127.4 and 318.4 per 100,000, respectively.

There was a significant increase in the ASIRs among women aged 70-74 (percentage change of 51% in 2007-2014) and 75-79 (39% in 2007-2013) over the recorded years. 

*ASIR in Japan*

**Figure 2:**
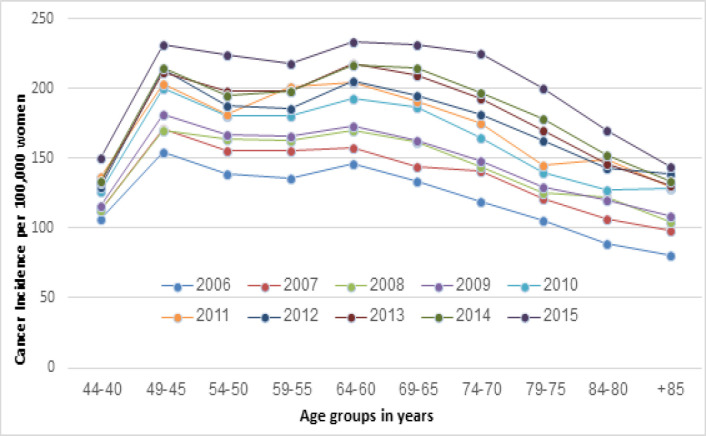
Age-Specific Incidence Rates (ASIRs) (per 100,000) of Japanese Women from 2006-2015 (National Cancer Center Japan, 2018)

The ASIRs of Japanese women are shown in Figure 2. There was a sharp rise between ages 40-44 and 45-49 years in all observed years, with 65% more breast cancers found in women aged 45-49 years compared to those aged 40-44 years in 2012. The highest BCI rate was seen in the age group 45-49 and the second highest incidence peak was observed in the age group 60-64 years. Since 2013, there was a slight change in this trend, with the BCI becoming slightly higher in women aged 60-64 than in women aged 45-49 (Figure 2). The BCI did not increase with age for Japanese women as seen in Australian women, but instead declined gradually after the age of 65 (Figure 2). 

During the same period, the ASIRs in Japanese women gradually increased each year in all age groups (Figure 2). The percentage change was most considerable among women aged 70-74 (88%), 75-79 (89%) and 80-84 years (90%), and least among women aged 40-44 years (40%).

*ASIRs in 2015*

**Figure 3:**
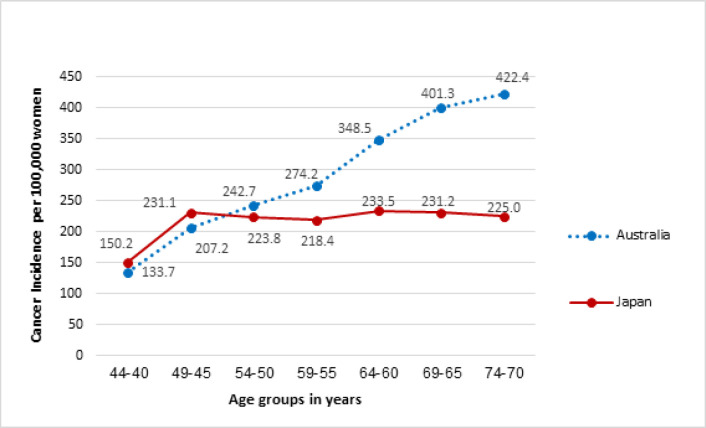
ASIR in Australia and Japan (per 100,000) in 2015 (AIHW, 2018a; National Cancer Center Japan, 2018)

The ASIRs of Australian women and Japanese women in 2015, which was the latest year for which data were available from all sources at the time of retrieval, are compared in Figure 3. Women aged 40-44 and 45-49 years showed almost the same ASIRs between countries (RRs= 1.12). In contrast, the ASIRs of women 50 years and older were significantly different and the difference increased with age. Japanese women aged 70-74 had 0.5 times the risk of breast cancer of Australian women in the same age group (RR=0.53).

*Screening participation and female population in Australia and Japan*

**Figure 4:**
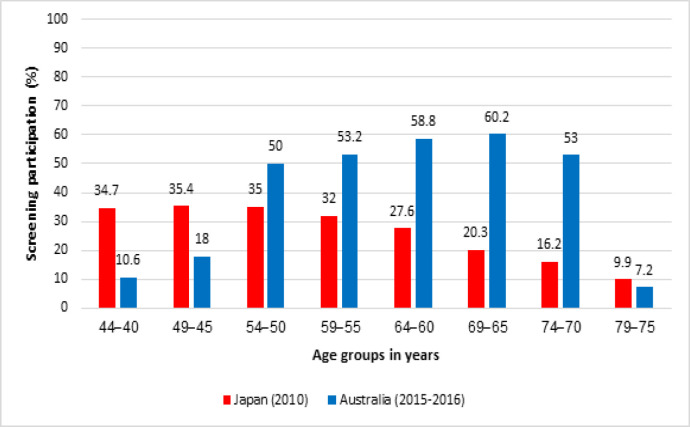
Screening Participation in Australia (2015-2016) and Japan (2010) by Age (Ministry of Health Labour and Welfare Japan, 2008; AIHW, 2018b)

Screening participation rates by age group between Australia (2015-2016) and Japan (2010) are compared in Figure 4. In Australia, the participation increases with age between 65-69 and then decreases from 70 years The participation for women aged 40-49 (11%) was significantly less than women aged 50-54 (50%) (AIHW, 2018b). In Japan, participation rates were higher for women aged 40-49 than those 50 and over, and then gradually decreases with age (Ministry of Health Labour and Welfare Japan, 2010). 

**Figure 5:**
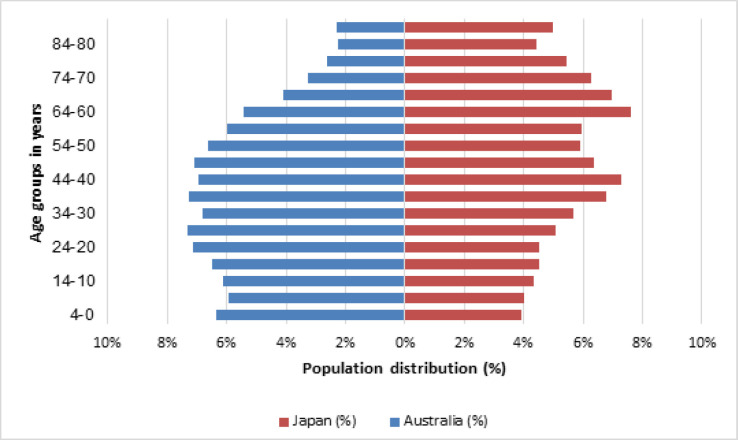
Female Population by Age in Australia (2010) and Japan (2013). (ABS, 2018; Statistics of Japan, 2015)

Female population by age is shown in Figure 5. Australian Bureau of Statistics (ABS) reported that the highest percentage of the female population among screening age in Australia was in women aged 40-44 (7.0%) and 45-49 (7.1%) (Australian Breau of Statistics [ABS], 2018). In Japan, however, statistics reported that women aged 40-49 (14%)and 60-69 (15%) made up the highest proportions of the total female population (Statistics of Japan, 2015). A declining trend was seen in women 70 years and over. 

## Discussion

This comparative analysis reported the patterns and trends of ASIRs in Australia and Japan. Age in particular, ageing is the predominant factor associated with BCI: more than 75% of breast cancer in Australia is found in women aged 50 and over (AIHW, 2012). In Australia ASIRs rose sharply from age 40 years and peaked at 65-69 years. The ASIR increased with age and gradually decreased after women reached 70, although it was higher than for women aged 40-49. The results provide evidence that the screening target for Australian women is appropriate to ensure that women at higher risk of breast cancer are recruited. However, it is important to acknowledge the multicultural nature of the Australian data. The previous study reported that ASIRs of migrants do not exhibit the same characteristics as those in either their country of origin or their destination country (Kaucher et al., 2018; Bhargava et al., 2019; Van Hemelrijck et al., 2019; Woods et al., 2019). Migrant Japanese women who have lived in the U.S. for more than a decade and several generations after migration increased their risk of breast cancer and thus increased their BCI rates (Ziegler et al., 1993). The impact of how ethnicity, including Japanese women living in Australia, affects on BCI rates requires further investigation. 

In Japan, previous studies have shown that BCI rates have been increasing over the years (Ohuchi et al., 2009; Matsuda et al., 2010; National Cancer Centre Japan, 2014) and the results of this analysis support their findings. From 2006-2015, increases in ASIR were found across all age groups. The results also demonstrated two peak ages of ASIR (45-49 and 60-64), and decline in ASIR with age, which is consistent with a previous study (Leong et al., 2010). In addition, this analysis demonstrated that the ASIRs of women aged 45-49 was higher than that of women over 50. Active recruitment of women aged 40 years and over into the national screening scheme is therefore appropriate for women living in Japan.

Comparison of the ASIRs of Australian and Japanese women provided new insight. Although previous studies reported that women in East Asian countries, including Japan, aged 40-49 had relatively high ASIRs and Australian women aged 40-49 had relatively low ASIRs (Ohuchi et al., 2009; Leong et al., 2010), this comparative analysis found that the ASIRs of women aged 40-49 in both Australia and Japan were very similar. Women aged 50 and over showed increased ASIRs in Australia while the trend in Japan showed a decline, which created the impression of different ASIRs among women aged 40-49 in each country. The difference in ASIRs between Australia and Japan became pronounced after the age of 50, due to a range of potential factors. 

*Factors contributing to differences in ASIRs*

The difference may be due to a number of potential contributing factors (Sitas et al., 2012). Potential factors such as screening participation rates, the age-specific distribution of the female population, number of mammographic views, and breast density are presented in this study, as discussed below.

Screening participation rates and ASIRs showed the same trends: in Japan both participation and BCI rates were higher for women aged 40-49 than those 50 and over. In Australia, participation rates were lower for women 40-49 than women 50 and over (Ministry of Health Labour and Welfare Japan, 2010; AIHW, 2018). The variation in participation most likely results from differences in recruitment into screening for women 40-49. With higher participation of Australian women aged 40-49 years in screening, an increased number of early stage cancers could be expected at a younger age. 

The Age distribution of the female population was similar to the trend in ASIRs in Japan but not in Australia. The highest percentage of the female population in Australia was found in women aged 40-49, and decreased with age, while ASIRs was lowest in women aged 40-49 and increased with age.

Two-view mammography has been found to detect more cancers than single mammography view (Wald et al., 1995) and might be contributing to the higher BCI rates of Australian women over 50 compared to Japanese women. For all Australian women and Japanese women aged 40-49, the cranio-caudal (CC) and medio-lateral oblique (MLO) are standard views taken in screening mammography. However, Japanese women 50 and over receive only the MLO view of each breast (Ministry of Health Labour and Welfare Japan, 2008; National Cancer Centre Japan, 2014; Tsunoda, 2014). Breast cancer screening in Japan began in 1987 using CBE (clinical breast examination) (National Cancer Centre Japan, 2014). Since 2000, MLO single view mammography screening has been implemented in combination with CBE for women aged 50 and older (National Cancer Centre Japan, 2014). In 2004, the screening guidelines were reviewed by the government and the target population was extended to women aged 40 to 49 years using 2-view mammography. However, the screening views for women aged 50 and older remained the same to minimise radiation exposure (National Cancer Centre Japan, 2014). A radiation risk-to-screening benefit evaluation model for 2-view screening for Japanese women aged 40-49 and single view mammography for those aged 50 and over showed that the benefits outweighed the radiation risks (Iinuma, 2012). 

Finally, increased breast density in women under 50 can negatively impact on breast cancer detection (Boyd et al., 2005). The low to medium ASIRs of younger women in this analysis may have resulted from masking of signs of early cancers by dense fibroglandular tissues (Boyd et al., 2005). The higher ASIRs of Australian women aged 50 and over, on the other hand, could be due to lower breast density, which increases the potential for breast cancer detection. 

*Limitations*

Several limitations in this comparative analysis should be noted. First, the ASIRs data used in this analysis is a combination of cancers detected by screening and diagnostic tests, therefore, the data is not a reflection of cancer detection by screening programs. Second, the stage at which the breast cancers were detected was unknown. If it was detected at a later stage of development, the age at which cancer started to develop could be younger than the age reported. This significantly impacts on the age contribution of the detection and the target population for screening programs. Third, the impact of the multicultural nature of Australian population on BCI data is unknown. Finally, there were differences in the quality of the data in Australia and Japan in relation, for instance, to the methods and modellings used to estimate ASIRs. 

*Further research*

This analysis supports the differing screening target of Australia and Japan. However, it is important to investigate the ASIRs for migrant women, including Japanese women living in Australia to ensure that Japanese women who migrate to Australia are appropriately covered by the Australian screening target age groups. Second, the inclusion of women aged 40-49 in the screening target in Australia requires further research since the results showed that the ASIRs in Australia and Japan were similar. It is crucial that breast density be considered to minimise the masking effect in screening mammography. Breast density distributions of Japanese women, Australian women, and Japanese women who have migrated to Australia were reported to be significantly different (Mizukoshi et al., 2019). The influence of duration of migration on breast density requires further investigation. Additionally, high mammographic breast density among women aged 40-49 needs to be taken into consideration (Mizukoshi et al., 2019). 

In conclusion, the analysis has emphasised the importance of the screening target age of each country and suggests that screening age range of Australian and Japanese national breast cancer screening guidelines provide effective coverage of ASIR peak ages for women in their home countries. The ASIRs of women aged 40-49 in Australia and Japan showed very similar results, but screening target age regarding the inclusion of women aged 40-49 in the targets were found to be inconsistent. Further evidence is required to confirm the inclusion of women aged 40-49 years in the screening target and the BCI rates of post-migrant women in Australia to ensure that the groups of women with highest cancer incidence are appropriately covered in breast screening.
